# Effects of Phytoestrogens on the Developing Brain, Gut Microbiota, and Risk for Neurobehavioral Disorders

**DOI:** 10.3389/fnut.2019.00142

**Published:** 2019-08-29

**Authors:** Cheryl S. Rosenfeld

**Affiliations:** ^1^Bond Life Sciences Center, University of Missouri, Columbia, MO, United States; ^2^MU Informatics Institute, University of Missouri, Columbia, MO, United States; ^3^Thompson Center for Autism and Neurobehavioral Disorders, University of Missouri, Columbia, MO, United States; ^4^Biomedical Sciences, University of Missouri, Columbia, MO, United States; ^5^Genetics Area Program, University of Missouri, Columbia, MO, United States

**Keywords:** genistein, daidzein, microbiome, cognition, anxiety, autism, soy formula

## Abstract

Many pregnant and nursing women consume high amounts of soy and other plant products that contain phytoestrogens, such as genistein (GEN) and daidzein. Infants may also be provided soy based formulas. With their ability to bind and activate estrogen receptors (ESR) in the brain, such compounds can disrupt normal brain programming and lead to later neurobehavioral disruptions. However, other studies suggest that maternal consumption of soy and soy based formulas containing such phytoestrogens might lead to beneficial behavioral effects. Select gut microbes might also convert daidzein and to a lesser extent genistein to even more potent forms, e.g., equol derivatives. Thus, infant exposure to phytoestrogens may result in contrasting effects dependent upon the gut flora. It is also becoming apparent that consumption or exposure to these xenoestrogens may lead to gut dysbiosis. Phytoestrogen-induced changes in gut bacteria might in turn affect the brain through various mechanisms. This review will consider the evidence to date in rodent and other animal models and human epidemiological data as to whether developmental exposure to phytoestrogens, in particular genistein and daidzein, adversely or beneficially impact offspring neurobehavioral programming. Consideration will be given to potential mechanisms by which such compounds might affect neurobehavioral responses. A better understanding of effects perinatal exposure to phytoestrogen can exert on brain programming will permit pregnant women and those seeking to become pregnant to make better-educated choices. If phytoestrogen-induced gut dysbiosis contributes to neurobehavioral disruptions, remediation strategies may be designed to prevent such gut microbiota alterations and thereby improve neurobehavioral outcomes.

## Introduction

Fetuses and infants are exposed to phytoestrogens through the maternal diet. Neonates can also be exposed to such compounds through soy milk, which has shown in a surgence in usage. With their structural similarity to 17β-estradiol, these chemicals may bind and activate estrogen receptors (ESR) within the brain during the perinatal and adult periods. Consequently, phytoestrogens might disrupt normal organizational-activational programming of the brain that is regulated by testosterone or neural conversion of testosterone to estrogen (1–4). With this primary concern in mind, animal model and human epidemiological studies have been initiated to examine how such class of chemicals affect neurobehavioral programming (5).

Phytoestrogens may be metabolized by gut microbes to bioactive forms with even greater potency (6). For instance, S-equol is one metabolite that results from intestinal bacterial metabolism of daidzein/daidzin (7). Population differences exist though in those possessing gut bacteria capable of this conversion compared to those who lack such resident flora with 60% of Asian individuals but only 25% of Western individuals harboring such gut bacteria able to synthesize S-equol (8). A study with 15 adult German individuals consuming a typical German diet revealed half of these individuals excreted S-equol at concentrations ranging from 8 to 128 ng/mL (9). After consumption of soy protein, elevated concentrations of GEN (820 ng/mL), daidzein (960 ng/mL), and S-equol (1,740 ng/mL) were detected. As reviewed by Yuan et al (10), with only 1/3 to ½ of the population as a whole able to metabolize daidzein to equol, not all individuals may reap the potential beneficial effects of soy isoflavones, such as prevention of cardiovascular diseases, osteoporosis, and select cancers. Those individuals lacking equol-generating bacteria tend to instead produce O-desmethylangolensin (ODMA). ODMA-producers have a higher predilection for obesity than equol-producers (11). In this way, gut bacteria can influence the how phytoestrogens might affect host systems, including neurobehavioral and metabolic responses.

Phytoestrogen consumption or exposure might also influence the gut microbe composition. Within Asian populations, increased consumption of soy is associated with increased number of equol-generating bacteria within the intestine (12). A similar study found that ingestion of daidzein positively correlated with greater abundance of *Asaccharobacter celatus* and *Slackia isoflavoniconvertens*, two equol producing bacteria (13). Other rodent and human studies suggest that genistein (GEN) or soy based foods impacts gut microorganisms (14–22). Alterations in bacteria residing in the gut or skewing to pathobionts (otherwise considered gut dysbiosis) may result in neurobehavioral changes, giving rise to the term the microbiome-gut-brain axis (23–26). Germ-free (GF) mice, which lack a gut microbiome, provide the first solid evidence of a connection between gut bacteria and host neurobehavioral responses. Impairment of the hypothalamic-pituitary-adrenal gland (HPA) axis results with the absence of normal gut bacteria, but bacterial reconstitution or fecal transplantation early on mitigates such effects (27). Moreover, GF mice are more anxious, less exploratory, and show cognitive and social deficits to provide a few examples of behavioral deficits characterized in this mouse model (25, 28–31). The microbiome-gut-brain axis term has been coined based on these above results (32, 33). It suggests that there is crosstalk between the gut microbiota and brain with bacteria within the intestines able to modulate neurobehavioral responses and in turn through the vagal nerve and other potential mechanisms, neural responses can influence the composition of gastrointestinal bacteria and the gut itself. Thus, phytoestrogen-induced changes in gut bacteria might be another way in which exposure to such compounds lead to later neurobehavioral disruptions.

**Figure 1:**
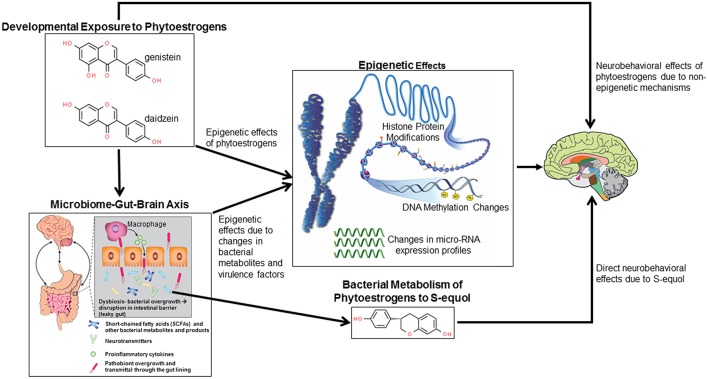
Potential mechanisms by which developmental exposure to GEN and other phytoestrogens might alter neurobehavioral programming. Such compounds might induce epigenetic and non-epigenetic changes in the brain, leading to later behavioral alterations. Phytoestrogens might alter gut microbial species leading to disruptions of the microbiome-gut-brain axis. Bacterial metabolites and virulence factors may induce host epigenetic modifications that alter neurobehavioral responses. Select bacteria may metabolize phytoestrogens to more potent metabolites, in particular S-equol, that can cause neurobehavioral alterations.

This review with thus consider the evidence to date that pre- and/or post-natal exposure to phytoestrogens with an emphasis on genistein, may cause later neurobehavioral deficits. Such effects might be due to direct binding of such chemicals to neural ESR, metabolism by gut microbes to even more potent compounds, or through phytoestrogen stimulated changes on gut bacterial populations. In relation to the latter possibility, there are several mechanisms by which gut bacterial shifts may affect host neural circuitry (23). Examples include shifts to pathobionts might lead to intestinal pathologies, such as increased gut leakiness or inflammation that could permit even more virulent organisms to penetrate through the gut barrier and induce systemic effects. Bacterial metabolites, including those that mimic host neurotransmitters, may be transmitted to the brain via the circulation or vagal nerve where they can disrupt normal homeostatic mechanisms. Should the bacteria enter into the circulation, they may then penetrate through the blood brain barrier and thereby gain direct access to neurons and glial cells. Phytoestrogen compounds and bacterial metabolites can also stimulate epigenetic changes in the brain, resulting in potential disruptive gene expression changes, which will be delved into further. These potential mechanisms by which developmental exposure to phytoestrogens can affect later neurobehavioral responses are summarized in Figure 1. Finally, open-ended questions and future directions in this area will be explored.

## Animal Model Studies Reporting Neurobehavioral Effects Following Gestational or Postnatal Exposure to GEN or Other Phytoestrogens

Rodent model studies reveal pre- and post-natal exposure to GEN can lead to reproductive (34, 35) and behavioral abnormalities such as increased defensive behaviors and demasculinization in male mice (36–38). Administration of GEN (either through diet-500 μg/g diet or gavage−20 to 75 mg/kg gavage) to pregnant Sprague-Dawley rats results in similar concentration of GEN aglycone in the fetal brain as identified in the maternal brain (39), strongly supporting the notion of relatively efficient placental transfer of this phytoestrogen. Another study with Sprague-Dawley rats also showed that ingested phytoestrogens can be transferred across the placenta and are concentrated in maternal milk, as evidenced by high infant plasma concentrations of these compounds (40). Thus, during pre- and post-natal life, GEN can pass through the blood brain barrier where it can result in direct neurobehavioral effects, as shown in Figure 1.

Male offspring derived from C57BL/6 dams supplemented with GEN (5 or 300 mg/kg) throughout gestation and lactation exhibit reduced anogenital distance, suggestive of outward feminization and decreased body mass with effects most pronounced in those receiving the low dose of GEN. Moreover, this group of males later showed reduced aggressive but increased defensive behaviors (36). CD1 dams exposed to GEN at 100 μg/g body weight from gestational day (GD) 11 to day 8 post-partum give rise to male offspring who as adult show increased anxiety and aggressive behaviors (37). These males had reduced number of positively stained cells in the amygdala neuronal cells staining positive for nitric-oxide synthase. A similar GEN exposure dose and period to Wistar rats resulted in sex-specific offspring effects with females displaying reduced novelty preference and exploration in an open field maze (41). On the other hand, developmental exposure to GEN (soy based diet, Purina 5001) from gestation through puberty has been suggested to abolish bisphenol A (BPA)-induced effects on anxiogenic behaviors in juvenile Wistar rats and restore normal sex differences in exploratory behavior in these animals (42). Using aromatase knockout (ArKO) mice, another study indicated that pre- and post-natal exposure to a phytoestrogen rich diet (containing 600 μg total dietary phytoestrogens, Harlan Teklad Global Diet 8604) is needed for normal brain sexual differentiation, namely sex differences in progestin receptor expression in the medal amygdala (43).

Exposure of Wistar rats from postnatal day (PND 0-3) to GEN (10 mg/kg body weight) advanced vaginal opening but reduced the number of kisspeptin immunoreactive fibers in the anteroventral periventricular nucleus (AVPV) and arcuate nucleus (ARC), two regions of the brain associated with the timing of pubertal onset and estrous cyclicity (44). This group also showed persistence in the number of multiple oocyte follicles. GEN (250 micrograms) administration on PND 1 and 2 to female and male rats resulted in females demonstrating similar number of AVPV neurons co-expressing tyrosine hydroxylase (TH) and estrogen receptor 1 (ESR1) as males, suggestive that this compound abolished normal sex differences in this brain region that would otherwise exist or defeminized these females (45).

In a recent study published by my laboratory, we showed that pre- and post-natal exposure of California mice (*Peromyscus californicus*), who are monogamous and biparental, to GEN (250 mg/kg feed weight) results in socio-communicative deficits when animals are weaned at 30 days of age (46). As detailed below, this recent paper also linked social and vocalization behaviors with GEN-induced gut microbiome and metabolome changes.

A study testing the effects of perinatal exposure to daidzein placed female Balb/cJ mice on a daidzein supplemented diet (200 mg/kg diet) throughout gestation and lactation and then examined anxiety-like and social behaviors and spatial learning and memory of adult male mice (47). Male mice developmentally exposed to daidzein had suppressed exploration but increased anxiogenic and aggressive behaviors. These males also exhibited increased social investigation of females and some improvements in spatial learning and memory. *Esr1* expression was increased in the bed nucleus of the stria terminalis, medial preoptic area, ARC, and central amygdala region but reduced in the lateral septum of these males. Collectively, these data suggest that early daidzein exposure masculinizes male-typical behaviors, which might be due to shifts in neural *Esr1* expression.

With their rapid developmental cycle, zebrafish (*Danio rerio*) have also been useful models in teasing apart the effects of early phytoestrogen exposure on brain development. Exposure of zebrafish embryos to GEN (10 μm) triggers apoptosis in the hindbrain and anterior spinal cord, which is not inhibited by co-administration with ICI 182,780 (48). *In vitro*, GEN can bind and activate ESR1, ESR2-A, and ESR2-B, and testing of transgenic ERE-luciferase fish reveals that this compound can activate estrogen pathways during the larval stages. Additionally, GEN stimulates ectopic expression of the aromatase-B gene in an ESR-dependent manner in the anterior brain. The findings from this study suggest that early GEN exposure of zebrafish may result in ESR-dependent and ESR-independent effects within the brain. Another study testing a range of GEN concentrations (0.25 to 1 × 10^−4^ M) found that this phytoestrogen stimulated EGFP expression in the brain of mosaic ESR zebrafish embryos (49). GEN-induced embryos displayed several pathological changes, including neural apoptosis, reduced heart rates, suppressed hatching times, reduced body length, and increased mortality in a dose-dependent manner. In those that survived, the higher doses of GEN also resulted in pericardial edema, yolk sac edema, and spinal kyphosis.

To test the effects of soy formula in a non-human primate, infant male rhesus macaques (*Macaca mulatta*) were provided cow's milk based formula, soy based formula, or soy formula with added manganese from birth to 4 months of age (50). Those reared on soy or soy + Mn based formula engaged in reduced play behavior and showed heightened affiliative clinging behavior in social interactions. Both groups also showed blunted wake cycles and shorter durations of daytime inactivity. The findings suggest that soy and Mn might interact to affect brain development.

## Gestational Exposure to Phytoestrogens and Feeding Infant Soy Formula Increase the Risk of Neurobehavioral Disorders in Children

Infants provided soy formula can have circulating concentrations of isoflavones (genistein, daidzein, and their glycosides) that are up to 13,000 to 22,000 higher than plasma estradiol, whereas, infants provided breast milk or cow milk have neglible amounts of these chemicals (51).

A study with 3,664 boys and 3,412 girls as part of the Avon Longitudinal Study of Parents and Children revealed that girls exposed during infancy to soy formula show reduced female-typical play behavior, but comparable disruptions were absent in boys (52). This same cohort revealed that girls fed soy products during infancy have an increased risk of early menarche, but this study only included a limited number of soy-exposed individuals and did not test specific mechanisms (53).

Based on medical records analyzed from the Simons Foundation Autism Research Initiative Simplex Collection that included data in infant formula and autism diagnostic score for 1,949 autistic children, a potential linkage between feeding infant soy formula and subsequent risk for autistic behaviors was found (54). Additional data from this cohort revealed that soy based formulas might contribute to febrile seizures in autistic boys and girls and a concurrent increased risk of epilepsy in these boys (55).

Other studies have found no differences in cognitive function and infants fed soy or breast milk. For instance, event-related potentials to speech sounds and behavioral measures were similar in infants fed either soy based formula or breast milk (56). While another study found differences in the electroencephalographic (EEG) activity in formula-fed vs. breast-fed infants, no differences were detected between those provided soy vs. milk formula (57). A similar study by this research group also found no differences in EEG spectral development between infants fed soy vs. milk-based formula (58). In a double-blind study, lactose free milk or lactose free soy based formulas did not improve difficulties in infant behaviors, such as fussiness, crying, or need for attention as reported by caregivers (59). Mental and psychomotor development indices also do not seem to differ between infants provided milk-based formula compared to soy based formula (60), but this study did find that infants who were breastfed showed improved cognitive development relative to both formula-fed groups.

## Effects of Phytoestrogens on the Gut Microbiome

Consumption of soy or exposure to phytoestrogens alters the gut microbiota denizens (14–22). Most current studies have focused on whether direct or adult exposure affects the gut microbiome, although there is every reason to believe that developmental exposure to such chemicals influences infant gut bacteria populations, which may be even more vulnerable during this initial colonization period than those of adults. This section will consider whether phytoestrogen exposure or soy consumption at any point in the lifespan affects gut microbial profiles.

Soy-fed neonatal White Dutch Landrace pigs show correlations between diet-responsive intestinal metabolites and gut microbes (61). Specifically, bacteria within the duodenum of sow-fed pigs have greater α-diversity (overall species diversity). In this intestinal region, soy-fed pigs possessed a greater percentage of Cyanobacteria. Extending from the duodenum to the jejunum and ileum, 77, 48, and 19 genera, respectively, were altered by diet. Associations existed with ileum metabolites, such as acylcarnitines and 3-aminoisobutyric acid, and diet-induced microbial shifts. Another study with White Dutch Landrace Duroc piglets showed that a soy formula diet affected the intestinal epithelial lining, microbial populations, and intestinal epithelial barrier as well as anti-inflammatory markers (62). The Peyer's patches within the ileum of sow-fed piglets possess larger lymphoid nodule size and second lymphatic nodules with prominent germinal centers, indicative of enhanced immune reaction. Soy fed piglets have increased expression of VE-cadherin and putrescine in the ileum. *Lactobacillaceae* spp. and *Clostria* spp. are significantly elevated but *Enterobacteriaceae* spp. decreased in soy-fed piglets.

The plasma metabolome of vegans consuming a soy-rich diet differs from that of omnivores, but the gut microbial profile between the two groups of individuals is surprisingly quite similar (16). Within the plasma metabolome, vegans had higher amounts of vitamins and other plant-derived compounds, including ascorbate, xanthine metabolites and derivatives of benzoate metabolism. This group also showed increased amount of metabolites associated with chlorogenic acid bacterial metabolism, such as hippurate, catechol sulfate and 3-hydroxyhippurate. While daidzein and genistein were predictably elevated in vegans relative to omnivores, equol concentrations did not differ between the two groups of individuals. The findings of this study suggest that a vegan or soy-rich diet might alter gut bacteria-derived metabolism but not necessarily the gut microbiota themselves.

Three particular studies in mice have shown that direct consumption of GEN and exposure during pre- or post-natal life is associated with gut microbiota changes, which may result in metabolic and cognitive changes (63–65). Firstly, in the study by Huang et al. (65), non-obese diabetic male and female mice were exposed orally to GEN (20 mg/kg body weight) from GD 7 to PND 21. Female GEN offspring had hyperglycemia (suggestive of type 1 diabetes), which was linked with decreased serum concentrations of inflammatory markers, interleukin (IL) 10, IgG2a, and IgM. Male GEN offspring only had decreased concentrations of IgG1. Contrasting sex differences were evident for other inflammatory markers with GEN females showing an overall increased in pro-inflammatory splenic cell counts, whereas T cell indices were decreased in males. By PND 90. GEN females demonstrated increased levels of Enterobacteriales, a bacteria associated with a pro-inflammatory response, and intestinal mRNA expression of the anti-inflammatory marker, α-defensin, was reduced at this timepoint. Taken together, this study suggests that perinatal exposure to GEN induces later sex-dependent differences in gut microbiota and associated host changes with females showing increased incidence of metabolic disorders and a heightened pro-inflammatory response but exposed males tending to have anti-inflammatory responses. This study, however, did not measure any indices of neurobehavioral function in these mice.

In another mouse study, adult C57Bl6 mice were provided a high fat diet (HFD) or a HFD containing GEN for 6 months (64). Those fed the latter diet gained less weight, had reduced serum triglycerides, and showed improve glucose tolerance relative to those fed the HFD alone. Correspondingly, mice consuming the HFD with GEN had modifications in the gut microbiota that were shifted to those producing less amounts of lipopolysaccharide (LPS, virulent factor typical of gram negative bacteria) and decreased expression of hepatic pro-inflammatory cytokines. Supplementation of GEN to those on the HFD also resulted in improved cognitive function. While the findings suggest that chronic exposure to GEN may reduce intestinal pathobionts, suppress hepatic inflammation, and improve cognitive ability, only potential correlations can be drawn between GEN-induced microbiota shifts and later host changes. This study also did not explore for potential sex differences or how microbiota shifts may result in beneficial host effects, such as through bacterial metabolite changes.

C57BL6 mice females were placed 3 week prior to breeding and throughout gestation and lactation one of four diets dies: (1) HFD, (2) HFD with low dose GEN (0.25 g/kg diet), (3) HFD with high dose GEN (0.6 g/kg diet), or (4) control diet (63). At weaning, female offspring derived from the HFD groups had reduced birth weights, exhibited glucose intolerance, and had elevated serum insulin, triacylglycerol (TG), and total cholesterol (TC) relative to the control group. In contrast, those derived from HFD + low GEN showed increased birth weight, improved glucose tolerance, and reduced fasting insulin concentrations compared to HFD offspring. TG and TC levels were reduced in HFD + high GEN group relative to HFD. Offspring derived from dams fed the HFD + low GEN diet also showed enrichment for *Bacteroides* and *Akkermansia*, whereas, *Rikenella* and *Rikenellaceae_RC9_ gut_group* were elevated in HFD + high GEN offspring. Taken together, the findings from this study suggest that maternal GEN might mitigate some of the negative HFD-induced metabolic effects, and changes in gut microbes might at least partially mediate such effects.

Metabolites produced by gut microbes may influence host function (23, 66–70). However, these above studies examining effects of GEN on the gut microbiome did not examine for changes in host or microbial metabolites.

A recent study with Southern white rhinoceros (SWR, *Ceratotherium simum simum)* suggested that dietary phytoestrogens might transform the gut microbiota composition, which may be another mechanism resulting in increased infertility in captive populations fed a diet enriched with such compounds (71). The bacterial profiles of SWR were compared to that of the greater one-horned rhinoceros (GOHR [*Rhinoceros unicornis*]), who consume a similar phytoestrogen rich diet but show high levels of fertility in captivity. Microbial communities differed between the two groups of rhinos with SWR possessing greater relative numbers of Bacteroidetes, while Firmicutes was elevated in GOHR. While the findings in this study suggest that consumption of a phytoestrogen-rich diet changes microbial populations and thereby affects host reproductive function, the small sample size used in this study population, especially for GOHR, could be of potential concern. Additionally, each rhinoceros species may have unique signature pattern of resident gut bacteria, phytoestrogen metabolism, and fecundity. This study also did not determine whether the infertility issues in SWR are central (hypothalamus or pituitary gland) vs. gonadal in origin. Notwithstanding, the findings provide further evidence in an endangered species that a phytoestrogen-rich diet can alter gut microbial composition with potential pathophysiological changes resulting in the host.

**Figure 2:**
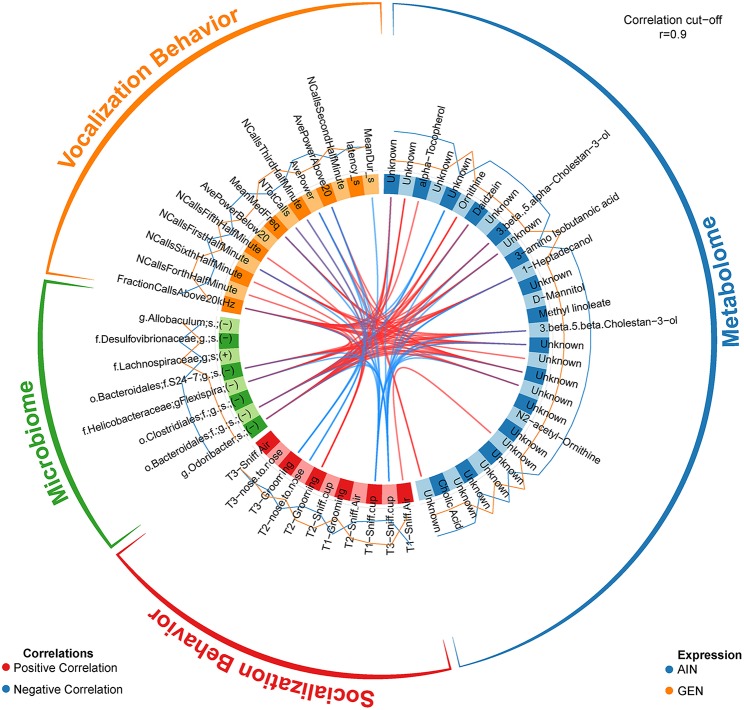
Circos correlations as determined with mixOmics analyses (72) for behavioral responses, gut microbiome, and gut metabolome changes in GEN exposed males vs. AIN (control) male California mice. Adapted from Marshall et al. (46) with permission from BioScientifica Limited.

**Figure 3:**
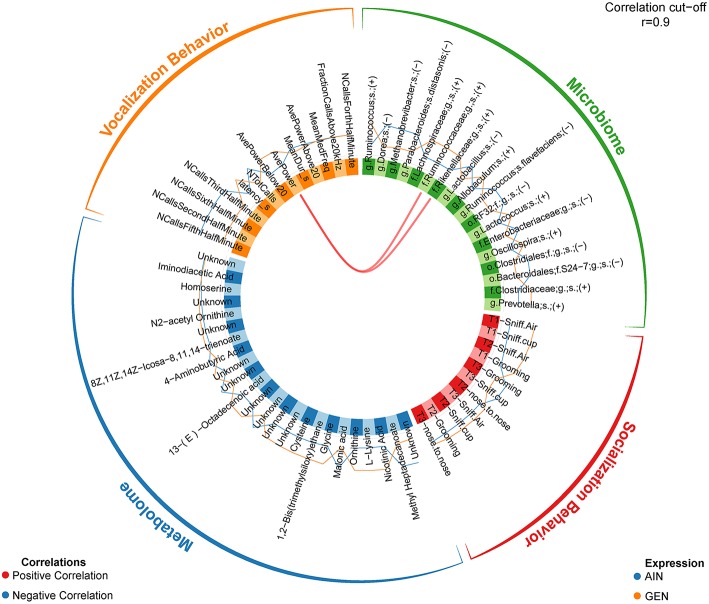
Circos correlations as determined with mixOmics analyses (72) for behavioral responses, gut microbiome, and gut metabolome changes in GEN exposed females vs. AIN (control) female California mice. Adapted from Marshall et al. (46) with permission from BioScientifica Limited.

In recent work from my laboratory, we discovered that developmental exposure of California mice to GEN (250 mg/kg feed weight) results in gut microbiome and metabolome changes, along with the neurobehavioral disruptions detailed above (46). By using mixOmics analyses (72), an R based program that allows for multiple data integration, we found several interactions between GEN-induced gut microbiome, metabolome, and socio-communicative behaviors (46). For instance, comparison of GEN males to AIN (control) males revealed that fraction of calls in the ultrasonic range (> 20 kHZ) was positively associated with daidzein, α-tocopherol, *Flexispira* spp., and *Odoribacter* spp. (Figure 2). Comparison of GEN exposed females to AIN females showed that average vocalization power positively correlated with *Rikenaceae* and *Ruminococcaceae* (Figure 3), both of these bacteria were elevated in females exposed to GEN. While findings with this informatics approach are interesting, they can only suggest correlation not causation. Even so, this informatics approach is likely to be useful in elucidating linkages between gut microbiome, gut metabolome, and neurobehavioral responses. Some follow-up studies can then be envisioned based on these results include treating control animals with the bacteria or metabolite that is linked with neurobehavioral deficits. Conversely, the effects of early supplementation of bacteria or metabolites that are reduced in GEN exposed animals might be tested to determine if such remediation strategies mitigate later neurobehavioral deficits.

The collective studies reveal the complexity of GEN and other phytoestrogens effects on gut microbiota communities, bacterial metabolites, and host responses. By altering the gut microbes and bacterial metabolite, GEN and other phytoestrogens might disrupt the microbiome-gut-brain axis, as shown in Figure 1. The net outcome of whether such changes lead to beneficial or detrimental effects is assumingly driven by a multitude of factors, including species, sex, when during the lifespan the exposure occurs, duration of exposure, other concurrent dietary factors, e.g., HFD, and host response being analyzed. Moreover, all of these current studies only reveal potential associations between gut microbiota/bacterial metabolite change and host effects. Actual causation studies would require transplanting the fecal microbiome from GEN or phytoestrogen exposed individuals into GF mice and then assessing whether similar phenotypic outcomes occur in these mice as identified in donors directly exposed to these xenoestrogens.

## Neurobehavioral Effects of Equol in Rodent Models and Human Studies

As reviewed in Setchell and Clerici (73), equol was first isolated in equine urine in 1932, and it was identified 50 years later in human urine as a metabolite of soy isoflavones, daidzin and daidzein. Equols are metabolites formed gut microorganism reduction of soy isoflavones (74, 75). In fact, many isoflavones must be metabolized in the gut by microbes in order to reach their full biological activity (76). Equol exists as a diasteroisomer with certain intestinal bacteria being enantiospecific in synthesizing exclusively the S-(-)equol enantiomer form (77, 78). This form demonstrates selective affinity for estrogen receptor beta (ESR2) (79).

The role of intestinal bacteria in converted isoflavones to equol is shown by the fact that GF animals provided a soy diet do not have detectable levels of equol in their urine (80). In humans who have had an ileostomy, where the ileum is attached to the abdominal wall to bypass the large intestine, have reduced production of microbial metabolites, including equol, dihydrogenistein, and ODMA in the urine (81). However, such patients are able to effectively deglycosylate isoflavonoid glucosides in the small intestine and have normal absorbtion of aglycone metabolites.

In humans, there is genetic heterogenity in equol production following soy isoflavone consumption (8). Importantly, only 30–50% of Western individuals produce equol, thus suggesting that only these individuals experience full metabolic benefits of dietary soy (8). While most equol-producing bacteria belong to the *Coriobacteriaceae* family, other bacteria can convert daidzein/daidzin (and to a lesser extent, GEN) to S-equol (75, 82, 83). While no current studies have examined the effects of developmental exposure on later neurobehavioral responses, S-equol has been associated, not only with postive metabolic effects, but also with neuroprotection based on *in vitro* and *in vivo* models (84–88). However, one study with ovariectomized rats indicated that daily treatment with S-equol did not positively affect learning and memory (89). In our recent study that tested the effects of S-equol (10 mg/kg body weight) in male and female C57BL6J mice provided a HFD, we found that this supplementation worsened aspects of HFD-induced metabolic disorders, as indicated by reduced physical activity in male and females, decraesed energy expenditure in males, and hyperglycemia/hyerinsulinemia (90). On the other hand, S-equol individuals had decreased anxiogenic and depression-like behaviors. Potentially analogous beneficial neural findings have been reported in women with equol producers and post-menopausal individuals consuming soy showing improved cognitive performance and emotional responses (91, 92). Ishikawata et al. (92) concludes that equol supplementation might be a useful adjuvant for menopausal women who are non-equol producers. However, additional studies are needed to determine the potential effects on mood-related symptoms and underpinning neural mechanisms. Thus, conversion of phytoestrogens to S-equol may result in neurobehavioral changes, as shown in Figure 1.

## Epigenetic Changes Induced by Phytoestrogens or Bacterial Metabolites

Phytoestrogens and bacterial changes induced by exposure to such compounds can lead to epigenetic changes within the host, as shown in Figure 1. While there is scant evidence showing phytoestrogens mediate epigenetic change in the brain, a wealth of data exists reveals these compounds alter the epigenome, especially DNA methylation changes, in other non-cancerous and cancerous cells and tissues. One of the earliest studies to show such effects placed male mice on a casein-based or GEN (300 mg/kg) diet for varying lengths of time (93). GEN treatment positively correlated with changes in prostate DNA methylation at CpG islands (93). Hypermethylation and subsequent silencing of the proto-oncogene H-Ras occurred in rats treated with the phytoestrogenic compounds, coumestrol or equol (94). One study suggested genistein may reverse hypermethylation of various cancer-promoting genes, including *p26*^*INK*4*a*^, *RARb*, and *MGMT* (95). However, this study was *in vitro* based with human esophageal squamous cell carcinoma, KYSE, and prostate carcinoma (LNCaP and PC3) cell lines.

Supplementation of GEN (250 mg/kg diet) to pregnant *a/a* dams carrying *A*^*vy*^*/a* (viable yellow mice) offspring resulted in a shift to more pseudoagouti offspring, who had increased methylation of CpG island sites within the *A*^*vy*^ IAP promoter site (96). Mice with the *A*^*vy*^ allele have an IAP inserted in the pseudoexon 1A region (97), resulting in *agouti* expression being controlled by the long terminal repeat (ltr) of the IAP proximal to the agouti gene, which is correlated with the degree of IAP methylation. Hence, *A*^*vy*^*/a* mice have coat colors ranging from yellow (hypomethylation of the *A*^*vy*^ IAP and who are prone to obesity and diabetes) to mottled (yellow with varying degrees of agouti patches) to completely pseudoagouti (full hypermethylation of the *A*^*vy*^ IAP who will be overall healthy). This same group also showed that concurrent developmental exposure to BPA and the same dose of GEN resulted in GEN mitigating the negative epigenetic effects of BPA on the *A*^*vy*^ gene with a subsequent reduction in the number of yellow coat-color *A*^*vy*^*/a* mice prone to develop metabolic disorders (98). Using a similar approach, however, we were not able to replicate these findings (99). Instead, we found that co-exposure during gestation to both BPA and GEN favored the birth of *a/a* compared to *A*^*vy*^*/a* mice. Besides altering the uterine transcriptome profile, neonatal exposure of female C57Bl6 mice to GEN (50 mg/kg from PND 1-5) caused dysrgegulation of genes involved in chromatin remodeling and altered transcriptional responses to subsequent glucocorticoid treatment (Dexamethasone 1 mg/kg from PND 5-21) (100).

GEN stimulates various epigenetic changes in human breast cancer cell lines. One study reported that in MDA-MB-231 human breast cancer cells, GEN resulted in hypomethylation in the promoter region of several tumor suppressor genes with concomitant increased expression of these beneficial genes (101). Similarly, in MCF-7 breast cancer cells, GEN suppressed methylation of BRCA1 and correspondingly inhibited cell proliferation (102). In MCF-7 and MDA-MB 231 breast cancer cell lines, GEN, daidzein, and equol caused a decrease in number of trimethylated histone protein marks, which tend to be associated with suppression of transcription, but an increase in number of acetylating histone protein marks, which remove histone proteins bound to DNA and thus increase gene transcription. The gene involved include *EZH2, BRCA1, ESR1, ESR2, SRC3*, and *P300* (103).

Comparison of genes that are epigenetically altered in the rat mammary gland to predict breast cancer patient survival using the Cancer Genomic Atlas (TCGA) revealed that two genes, *HPSE* and *RPS9*, were hypomethylated in the mammary gland of rats prepurtertally treated with GEN or GEN + BPA and were also associated with increased patient survival (104). These findings and the *in vitro* studies above provide strong evidence that at least in the mammary gland, GEN may induce beneficial DNA methylation and other epigenetic changes. Similarly, GEN and soy protein isolate (SPI) repress WNT signaling in the rat colon, which is associated with a reduction in pre-neoplastic lesions (105). Follow-up studies reveal that GEN and SPI appear to inhibit WNT signaling by methylating defined regions within *Sfrp2, Sfrp5*, and *Wnt5a* and by altering histone protein binding to DNA, as judged from an increase in histone deacetylase 3 (HDAC3), suppressing trimethylation of histone 3 lysine 9 (H3K9me3). Additionally, both treatments inhibit phosphorylation of histone H3 Serine 10 (H3S10P) (106).

A multitude of host effects can result from gut microbial changes. Bacterial metabolites and other products, such as LPS in Gram negative bacteria and peptidoglycans from Gram positive bacteria, can induce a variety of host epigenetic changes, as reviewed in Rosenfeld and Kapila (23), Bhat et al. (107), Alam et al. (108), Eshraghi et al. (109). Herein, we will consider a few examples.

Histone proteins that bind to DNA are subject to various epigenetic modifications, such as methylation, acetylation, sumoylation, and ubiquitination, and such post-translational modifications alter the affinity to which these proteins bind to DNA. Acetylation tends to pull the histone proteins away from DNA, resulting in increased gene activation, whereas, deacetylation of histone proteins is associated with increased histone protein binding and decreased gene transcription. Acetylation of histone proteins is regulated by two primary class of enzymes, histone aceytltransferases (HAT) that transfer acetyl groups to histone proteins and histone deacetylases (HDAC) that remove acetyl groups from these proteins. Several bacterial products can affect these two enzymes. Bacterial-derived short chain fatty acids (SCFA), mainly butyric acid (BA), propionic acid (PPA), and acetic acid act as HDAC inhibitors with BA being the most potent (110, 111). Other SCFA, such as lactate and pyruvate, may induce weak HDAC inhibitory responses (112–114).

In contrast, acetate, a byproduct of microbial fermentation of dietary fiber, up-regulates HAT substrate availability (26). Acetate supplementation appears to mitigate neuroglial activation and loss of cholinergic cells in rats with neuro-inflammation due to LPS treatment (115). These beneficial effects are likely due to increased acetylation of neural H3K9, H4K8, and H4K16 and acetate suppression of HDAC2. The metabolite, epigallocatechin-3-gallate (EGCG), primary component of green tea, also can increase acetylation of H3 and H4 and simultaneously inhibit HDAC and DNA methyltrasferase (DNMT) (116). Conversely, fisetin, a flavonoid found in fruits and vegetables activates HDAC but suppresses HAT (117).

Several gut microbiota synthesize a variety of water soluble B-vitamins, including folate or folic acid, biotin, vitamin B2, B6, and B12, that can promote methylation of DNA and histone proteins and induce other epigenetic changes (118). Folate, vitamins B2, B6, and B12 enter at various points into one-carbon metabolism, which leads to the synthesis of the common methyl donor, S-adenosyl methionine (SAM), which also acts as a substrate for DNMT (119). Thus, bacterial production of B- vitamins can alter host DNA methylation and potentially histone protein methylation profiles.

Human and animal studies provide further evidence that gut bacterial shifts lead to DNA methylation and subsequent gene expression changes in various host cells and tissues. A linkage exists in pregnant women between gut microbial profiles, namely for Firmicutes and Bacteroidetes, and leukocyte DNA methylation patterns for genes regulating lipid metabolism and obesity (120). Feeding mice a maternal methyl supplemented diet causes gut dysbiosis and associated changes in colonic mucosal DNA methylation and transcriptomic patterns, and colitis in resulting offspring (121).The presence of gut bacteria stimulates hypermethylation of CpG motifs in the *Tlr4*/*TLR4* gene, whereas in the percentage of methylation of CpG motifs in the 5′ region of the *Tlr4* gene becomes reduced within the large intestine of GF mice (122).

Other epimutations may result due to changes in gut flora, such as chromatin rearrangements, alterations in non-coding or micro (mi)RNAs, and RNA splicing factors (123). Colonization of GF mice with commensal gut microbes alters the miRNA expression profile within the ileum and colon, resulting in gene expression changes in these regions (124). Several miRNA, such as miR-143, miR-148a, miR-200b, miR-200c, and miR-378 are downregulated in the intestines of mice infected with *Listeria monocytogenes* (125). In contrast, commensal gut microbes, *Escherichia coli* and A4 bacteria inhibit the expression of miR-10a in antigen-presenting dendritic cells through toll-like receptors that induce an MyD88-dependent pathway (126). This miRNA is associated with induction of a variety of cancers and inflammatory bowel disease. Therefore, suppression of this gene by beneficial gut microbes may be a useful adjuvant treatment for both of these disorders.

Gut pathogens might induce widespread effects on host gene expression profiles by suppressing RNA polymerase II, an enzyme required for synthesis of coding and non-coding RNAs (127). Intriguingly, select endosymbiotic bacteria seem to produce small non-coding RNAs with the potential to exert cross-kingdom communication and affect host processes (128). As we delve deeper into the complex relationships that have evolved between host and resident microorganisms, more such complex inter-relationships with the potential to affect the host epigenome and gene profiles are likely to be discovered.

## Conclusions

The current animal and humans studies reveal that developmental exposure to GEN and other phytoestrogens can induce mixed effects on later neurobehavioral responses that are likely mediated by a variety of processes (summarized in Figure 1). Offspring sex, timing and duration of exposure, and phytoestrogen examined are assumingly a few variable that determines whether the effects of these compounds is beneficial or detrimental to neurobehavioral responses, as well as the types of behaviors that are affected by early exposure to such compounds. In male rodents, exposure to phytoestrogens alone tends to induce an increase in anxiogenic behaviors but reduces exploratory behaviors (37, 47). Thus, it is not clear how to interpret the finding that co-exposure GEN and BPA suppresses anxiety-like behaviors that occur due to BPA exposure alone (42). The findings though suggest that other dietary components need to be considered when assessing potential neurobehavioral disturbances due to phytoestrogens. Such is the case also when mice are co-exposed to GEN and a HFD in that former seems to alleviate the disease-promoting effects of the latter (63, 64).

Similarly, the human studies have yielded vastly different findings with some reports indicating play behavior in girls is affected by early phytoestrogen exposure (52). Other studies implicate early exposure to such compounds in affecting risk that sons will develop autism and other behavioral disorders (54, 55), although it is clear that boys are already at a least 3:1 greater risk for these disorders than girls (129). Additional reports indicate babies provide soy formula do not show different behavioral patterns that those provide other types of formula (56–60). Taken together, the current human data do not provide a clear picture as to whether phytoestrogens or soy formula or consumption of such products by expecting mothers jeopardizes offspring brain development and risk for neurobehavioral disorders. Larger cohort studies are needed that also screen for maternal intake of soy and other products containing phytoestrogens and then link such maternal intake and/or feeding of soy formula with various behavioral trajectories in sons and daughters. The current studies though provide some ideas as to which behaviors should be considered in these additional epidemiological studies.

Such behavioral changes might be due to direct effects on the brain. Developmental exposure to GEN results in vacuolization of oxytocin neurons in the neonatal mouse hypothalamus (130). Brain nitrergic and vasopressinergic circuitry systems in rodents and Japanese quail might also be vulnerable to GEN (131–133). GEN exposure might also impede the gonadotropin releasing hormone network and hypothalamic-pituitary-adrenal axis (134–139).

Besides inducing direct effects on brain programming, early exposure to phytoestrogens, namely GEN, may alter resident gut flora populations that could in turn affect neurobehavioral functions through the microbiome-gut-brain axis. As with direct effects on the brain, gut microbiome alterations likely depend upon type of phytoestrogen exposure, host species examined, and time and duration of exposure to phytoestrogens (14–21, 61, 62). GEN-induced changes have been previously linked to changes in host cardiometabolic responses (17). Yet, studies examining the effects of phytoestrogens on gut microbial profiles and host responses can only establish correlation between these types of changes. To establish that such gut microbe changes actually cause host alterations, fecal microbial transfer from phytoestrogen-exposed individuals into GF mice that lack resident gut microbiota and who have not been exposed to such chemicals is required. If the gut flora alterations due to phytoestrogens lead to neurobehavioral disruptions, then GF mice transplanted with fecal material from exposed mice should demonstrate similar phenotypic changes. Such was the case when samples from offspring exposed to maternal HFD were transferred to GF mice who went on to display similar social deficits as the donor mice derived from dams on the HFD (140).

The resident gut microorganisms may also convert ingested or exposed phytoestrogens to even more potent forms, such as conversion of daidzein/daidzin to equol derivatives. While most rodent models have bacteria capable of this metabolism, human populations tend to be segrated into equol-producers, mainly from Asian countries, and non-equol producers, other geographical regions (8). Individuals lacking equol-generating bacteria tend to instead produce ODMA, and these individuals have a higher predilection for obesity than equol-producers (11). The current animal and human studies suggest that direct exposure to equol may yield beneficial cognitive and emotional effects. However, it remains to be ascertained how early exposure to this bacterial metabolite affects later neurobehavioral outcomes.

Phytoestrogen-induced changes in gut bacteria may affect other gut bacterial metabolites. As detailed above, soy-fed neonatal White Dutch Landrace pigs show linkages between diet-responsive intestinal metabolites and gut microbes (61). Such metabolite changes may be transmitted through the vagal nerve or systemic circulation to affect neural function. To establish actual causation between such bacterial metabolites and neurobehavioral changes, metabolites identified to be enriched due to phytoestrogen exposure would need to be administered to control dams and offspring neurobehavioral responses examined throughout the lifespan. This approach was done with 4-ethylphenylsulfate (4-EPS), a metabolite shown to be elevated in maternal immune activation mouse model for ASD, which resulted in autistic-like behaviors in control mice (141).

Another mechanism to dissect neurobehavioral effects directly due to phytoestrogen exposure vs. those mediated by the gut microbiome-metabolome, intracerebroventricular (ICV) injection with radioactive labeled phytoestrogens might be performed in neonatal animals, as has been done for other estrogenic compounds (142–144). This method, however, is likely not feasible in fetal mice.

The current studies also reveal that phytoestrogens might alter neurobehavioral responses through epigenetic changes, including DNA methylation, histone protein modifications, and alteration in miRNA profiles. Further work is thus needed to examine whether such compounds directly affect the enzymes mediating these epigenetic modifications, including DNMT, putative DNA demethylases, HDAC, and HAT. Phytoestrogen-induced gut dysbiosis and resulting changes in bacterial metabolites and virulence factors may also impact the host epigenome. This could be due to alteration in substrates, such as acetate or B-vitamins, required for such epigenetic modifications and/or by affecting host enzymes catalyzing these reactions. Of these, bacterial-derived SCFA, in particular BA, can significantly suppress HDAC, which would result in acetyl groups remaining on the histone proteins and separated away from their associated DNA strand with their promoter region being available for transcription factor binding and increasing gene transcription. Direct supplementation of such bacterial metabolites or challenging the host with bacterial virulence factors, such as LPS, will be useful in teasing apart how bacterial fluctuations affects the host epigenome and downstream CNS dysfunction and risk for other host diseases.

In conclusion, developmental exposure to phytoestrogens and potential effects on neurobehavioral function may be thought of as a see-saw with the ultimate outcome dependent on other contributory factors that can from the other side push the board and ultimate responses in a downward or upward direction. Such factors include offspring sex, type, duration, and timing of phytoestrogen exposure, other dietary components or endocrine disrupting chemicals, species examined, and behavioral changes considered to list just a few examples. In the middle of the board rests the gut microbiome, dramatically influencing in a variety of ways the ultimate outcome.

## Author Contributions

CR researched the area, wrote the manuscript, and revised it based on the Editor and Reviewers suggestions.

### Conflict of Interest Statement

The author declares that the research was conducted in the absence of any commercial or financial relationships that could be construed as a potential conflict of interest.
